# TRMA syndrome with a severe phenotype, cerebral infarction, and novel compound heterozygous *SLC19A2* mutation: a case report

**DOI:** 10.1186/s12887-019-1608-2

**Published:** 2019-07-11

**Authors:** Xin Li, Qing Cheng, Yu Ding, Qun Li, Ruen Yao, Jian Wang, Xiumin Wang

**Affiliations:** 1 Department of Endocrinology and Metabolism, Shanghai Children’s Medical Center, Shanghai Jiaotong University School of Medicine, 1678 Dongfang Road, Pudong New Area, Shanghai, 200127 China; 2 Department of Medical Genetics and Molecular Diagnostics, Shanghai Children’s Medical Center, Shanghai Jiaotong University School of Medicine, Shanghai, 200127 China

**Keywords:** Thiamine-responsive megaloblastic anemia, *SLC19A2* gene, Novel mutation, Diabetes, Deafness

## Abstract

**Background:**

Thiamine-responsive megaloblastic anemia (TRMA) is a rare autosomal recessive inherited disease characterized by the clinical triad of megaloblastic anemia, sensorineural deafness, and diabetes mellitus. To date, only 100 cases of TRMA have been reported in the world.

**Case presentation:**

Here, we describe a six-year-old boy with diabetes mellitus, anemia, and deafness. Additionally, he presented with thrombocytopenia, leukopenia, horizontal nystagmus, hepatomegaly, short stature, ventricular premature beat (VPB), and cerebral infarction. DNA sequencing revealed a novel compound heterozygous mutation in the *SLC19A2* gene: (1) a duplication c.405dupA, p.Ala136Serfs*3 (heterozygous) and (2) a nucleotide deletion c.903delG p.Trp301Cysfs*13 (heterozygous). The patient was diagnosed with a typical TRMA.

**Conclusion:**

Novel mutations in the *SLC19A2* gene have been identified, expanding the mutation spectrum of the *SLC19A2* gene. For the first time, VPB and cerebral infarction have been identified in patients with TRMA syndrome, providing a new understanding of the phenotype.

## Introduction

Thiamine-responsive megaloblastic anemia (TRMA, OMIM 249270)—also called Rogers Syndrome—was first described by Rogers LE et al. [1] in 1969. It is caused by mutations in the *SLC19A2* gene (solute carrier family 19 member 2), which is located on the chromosome 1q23.2–23.3 (OMIM 603941), and encodes the high affinity thiamine transporter-1 (THTR-1) [2]. Patients with TRMA are characterized by diabetes, megaloblastic anemia, and progressive sensorineural hearing loss during early childhood [3], and oral thiamine administration can effectively alleviate the symptoms of diabetes and anemia [4]. To date, only 100 cases of TRMA syndrome have been reported worldwide [5]. Here, we introduce the clinical features, diagnosis, and treatment of a Chinese boy with a typical TRMA syndrome.

## Case presentation

### Clinical data

A six-year-old boy was referred to the Department of Endocrinology at Shanghai Children’s Medical Center, Shanghai Jiaotong University School of Medicine (Shanghai, China) for being lethargic and pale for 1 week. During this time, he had developed polydipsia, polyuria (>10 times of the daily amount), polyphagia, and weight loss (4 kg in 1 week); however he had no fever, cough, vomiting, or diarrhea.

His medical history showed that he had cerebral infarction with paralysis of the left arm and ventricular premature beat (VPB) without infection symptoms at the age of 9 months, bilateral hearing loss at the age of 1 year, and cochlear implant surgery at the age of one and a half years. When he was 4 years old, he was admitted to the local hospital because of listlessness, and his medical condition was diagnosed as diabetes mellitus. However, he did not receive any insulin therapy, but his blood glucose level was measured every 2–3 months. He was born at full term following an uneventful pregnancy to non-consanguineous parents of Chinese descent. He had no relevant family history of diabetes or deafness.

On examination, he was found to be 112.5 cm tall (−1SD: 111.1 cm, −2SD: 115.8 cm), and he weighed 17 kg (−2SD, 17.27 kg). He had horizontal nystagmus and was sensitive to light. His heartbeat showed arrhythmia, but without any obvious arrhythmic sound. Furthermore, he had hepatomegaly 2 cm below the rib line. No other physical abnormalities were detected.

**Fig. 1:**
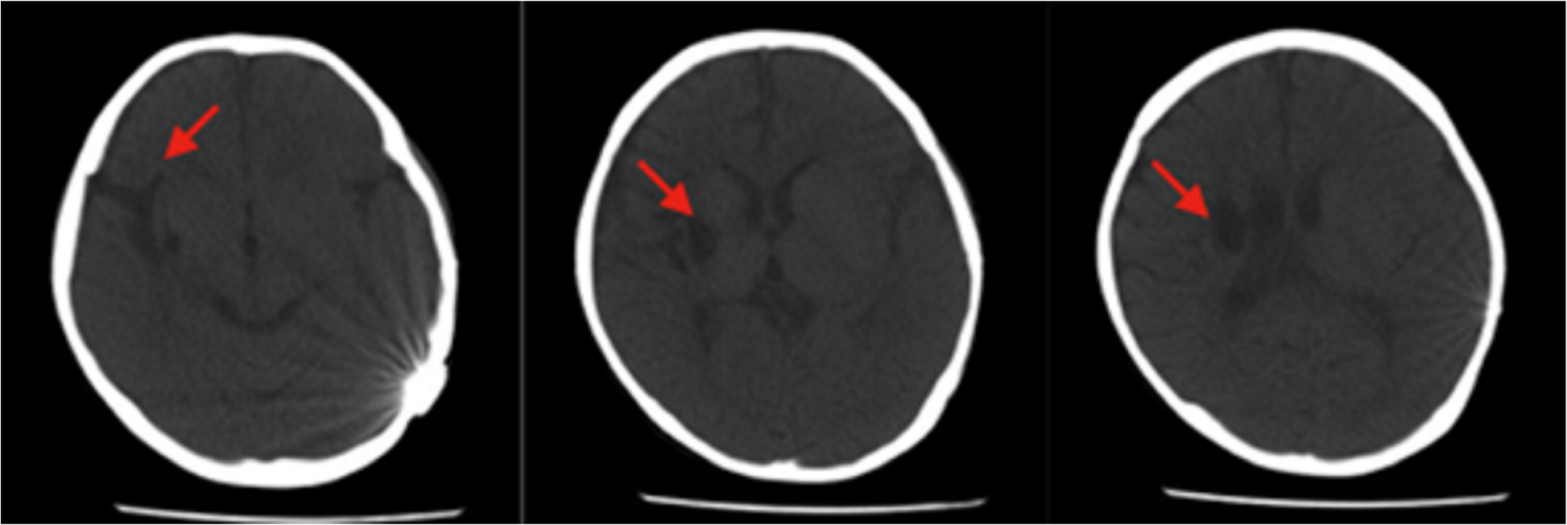
The head CT scan revealed right basal ganglia softening (indicated by an arrow) and right hemisphere cerebral atrophy

The electrocardiogram (ECG) showed an ectopic rhythm (VPB) and QT extension, and echocardiography showed a slightly enlarged left atrium and left ventricle (LA = 2.57 cm, LVDD = 4.27 cm; the limit of a six-year-old child: LA = 2.15 cm, LVDD = 3.89 cm) with normal left ventricular systolic function. A homogeneous or heterogeneous pancreas and two enlarged kidneys with diffusely enhanced medullas were observed in the abdominal ultrasound. Head CT scan revealed atrophy of the right cerebral hemisphere, and a softening at the right side of the basal ganglia (Fig. 1).

The patient had hyperglycemia (serum glucose: 26.5 mmol/L) and diabetic ketoacidosis (ECO_2_: 16.0 mmol/L, serum and urine ketones: 4+). His serum insulin and C peptide levels were below normal (serum insulin: 0.1 uIU/mL, normal range 1.9–23; C peptide: 0.64 ng/ml, normal range 1.1–4.4), while his glycated albumin (GA) and glycosylated hemoglobin (HbA1c) levels were elevated (GA: 46.6%, normal range 11–16%; HbA1c: 74 mmol/mol, normal range 18–40). Antibodies pertaining to diabetes, such as glutamic acid decarboxylase antibody (GADA), islet cell antibody (ICA), and insulin antibody (IAA) were absent in his blood. He was also found to have leukopenia (Neutrophils: 1.0 × 10^9^/L, with WBC: 3.1 × 10^9^/L), thrombocytopenia (Platelets: 52 × 10^9^/L), and anemia (Hb: 62 g/L, MCV: 98.0 fl, MCH: 31.6 pg, MCHC: 32%).

As he had anemia, we performed some examination to identify its type. Vitamin B12 and folic acid levels were not low at all (1333.00 ng/L, normal range: 197–866; 12.90 μg/L, normal range: 3.1–17.5, respectively). In addition, the levels of serum iron and serum iron saturation were above normal (31.1 μmol/L, normal range: 6.6–26; 80.2%, normal range: 20–55%, respectively), while the levels of unsaturated iron binding capacity, total iron binding capacity, and transferrin were below normal (7.7 μmol/L, normal range: 20–62; 38.8 μmol/L, normal range: 54–77; 1.60 g/L, normal range: 2–3.6, respectively). Bone marrow cytology showed active bone marrow hyperplasia, and active iron in the red blood cells (the positive rate of iron staining was 82%, and a few were suspected as ring sideroblasts). However, there were no obvious abnormalities in his hepatic, renal, hematopoietic and thyroid functions and urinary amylase levels.

### Next generation sequencing, and data analysis

Genomic DNAs of the patient and his parents were isolated from 2 ml peripheral blood samples collected from the cubital veins using a QIAamp Blood DNA Mini Kit® (Qiagen GmbH, Hilden, Germany). A total of 3 μg of the patient DNA was sheared to fragments of 150–200 bp using a Covarias® M220 Ultrasonicator system (Covaris, Inc. Woburn, MA, U.S.). An adapter-ligated library was generated with Agilent SureSelect Target Enrichment System (Agilent Technologies, Inc., Santa Clara, CA, U.S.) according to the manufacturer’s instructions. Capture library was prepared using an XT Inherited Disease Panel (cat. No.: 5190–7519, Agilent Technologies, Inc.), and comprised 2742 genes. Clusters were then generated through isothermal bridge amplification using an Illumina cBot station, and sequencing was performed with an Illumina HiSeq 2500 System (Illumina, Inc., San Diego, CA, U.S.).

Base calling, and assessment of the sequence read quality were performed using Illumina HCS 2.2.58 software (Illumina, Inc.) for the Illumina HiSeq 2000 system, which included new versions of HiSeq control software and Real Time Analysis. Alignment of the sequence reads against a reference human genome (Human 37.3; SNP135) was performed using NextGENe® (SoftGenetics LLC, State College, PA, U.S.). All single nucleotide variants (SNVs) and indels were saved in a VCF format file and uploaded to be processed with Ingenuity® Variant Analysis™ (Ingenuity Systems, Mountain View, CA, U.S.) for biological analysis and interpretation.

The primers for amplification of the *SLC19A2* gene (GenBank accession no.: NM_006996.2) were designed using UCSC ExonPrimer online software (http://genome.ucsc.edu/index.html) and synthesized by Map Biotechnology, Co., Ltd., Shanghai, China. The primers designed for exon 3 were as follows: forward 5′-TCGCCAGAGGGGATAAATG-3′, and reverse 5′-AGTCATAGTCCTGCTCCACTTG-3′. The primers designed for exon 2_1 were as follows: forward 5′- TCTGAACTGCTGTTGTCAAGG-3′, and reverse 5′- AGCCTGCCACTGAGACAAG − 3′. The exon and exon-intron boundaries were amplified using polymerase chain reaction (Takara Biotechnology, Co., Ltd., Dalian, China). The resulting DNA was sequenced with the forward and reverse primers using an ABI3730XL sequencer (Applied Biosystems; Thermo Fisher Scientific, Inc., Waltham, MA, U.S.). The sequence data were analyzed using Mutation Surveyor® software version 4.0.4 (SoftGenetics, LLC.).

**Fig. 2:**
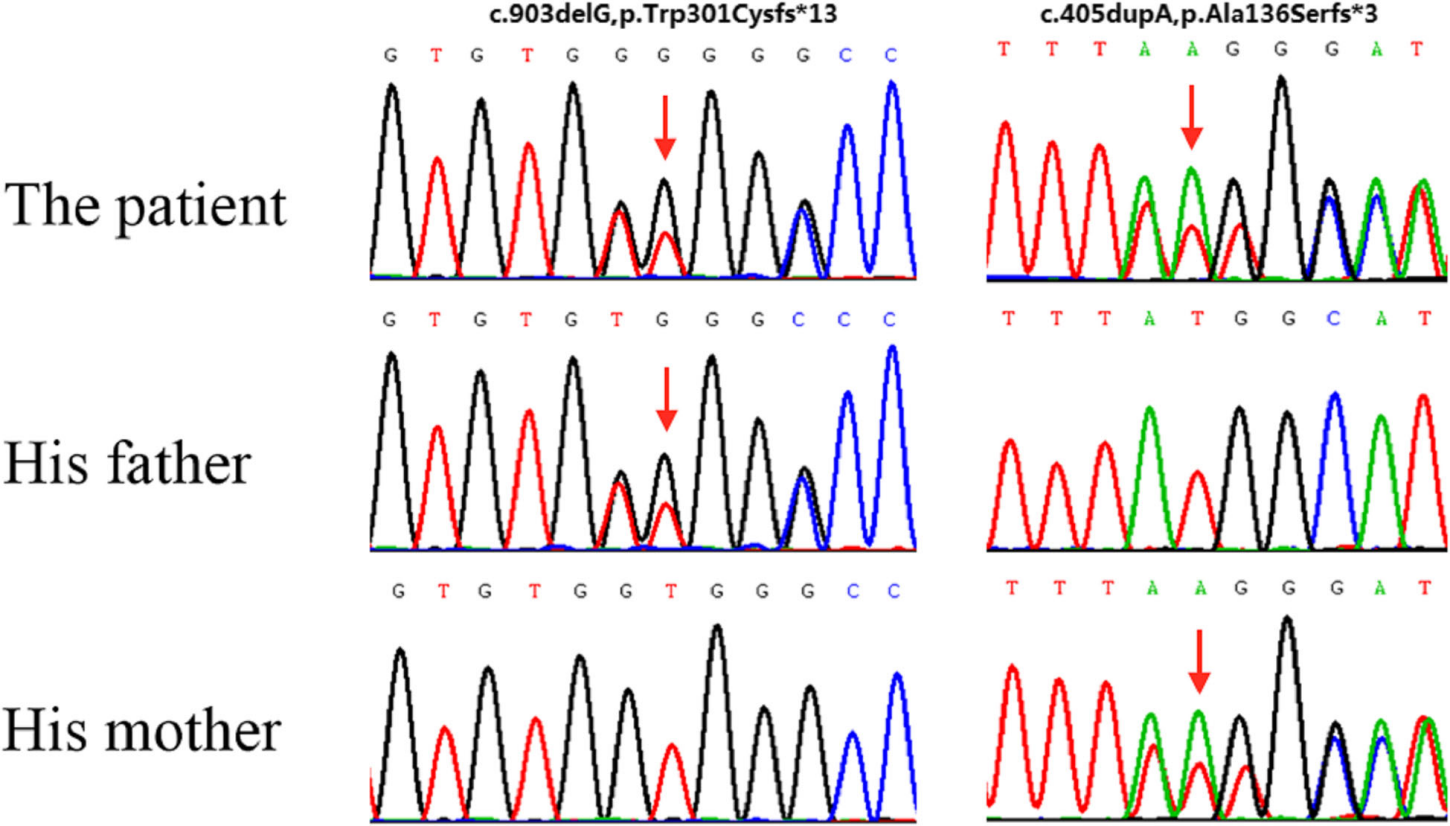
Novel mutations were identified in the *SLC19A2* gene. Sequences showed two heterozygous mutation (c.903delG p.Trp301Cysfs*13; c.405dupA, p.Ala136Serfs*3). His father carried c.903delG p.Trp301Cysfs*13 (heterozygous) and his mother carried c.405dupA, p.Ala136Serfs*3 (heterozygous).The red arrow indicates the mutation site

For a rapid and accurate clinical diagnosis, the patient was screened for causal variants using targeted DNA sequencing according to an in-house analysis protocol. Consequently, a novel compound heterozygous mutation in the *SLC19A2* gene of the patient was identified: a single nucleotide deletion (c.903delG) in exon 3, and a duplication (c.405dupA) in exon 2_1 (Fig. 2). The two frameshift variants do not exist in the current the genome Aggregation Database (gnomAD). Alamut software predicted that the mutation could cause premature termination during translation, resulting in the formation of a truncated THTR-1 protein. Therefore, we classified this compound mutation as a “pathogenicity variation.” Subsequently, the *SLC19A2* genes of the patient’s parents were analyzed using Sanger sequencing. Thereby, it was identified that the patient’s father carried c.903delG mutation (heterozygous) in the *SLC19A2* gene, and the patient’s mother carried c.405dupA mutation (heterozygous).

### Diagnosis, treatment, and follow-up

Based on the bone marrow cytology, genetic analysis, and clinical presentations, such as diabetes, anemia, and deafness, the patient was diagnosed to have TRMA syndrome. Consequently, he was treated with insulin (1 U/kg/day), thiamine (30 mg/ day), and propafenone (150 mg/day). Following thiamine administration, the patient’s blood glucose showed improved levels of thiamine (5–6 mmol/L), and his daily insulin requirements progressively decreased to 0.5 U/kg/day. In addition, the hemoglobin level in his blood gradually increased from 62 g/L to 112 g/L in 3.5 months, and his ECG reading turned to normal in 1 month.

## Discussion and conclusions

Thiamine, also known as vitamin B1, is widely found in the skeletal muscle, cardiac muscle, liver, kidney, and brain. Under physiological concentrations, thiamine is transported into the cell mainly by saturable, high affinity and low adhesion transporters THTR-1 and THTR-2, which are encoded by the *SLC19A2* and *SLC19A3* genes, respectively. After entering the cell, thiamine is converted to thiamine diphosphate (TDP). In the cytoplasm, TDP functions in the pentose phosphate pathway with transketolase (TK); in the mitochondria, it functions in the tricarboxylic acid cycle with α-ketoglutarate dehydrogenase (α -KGDH) and pyruvate dehydrogenase complex (PDHC). The *SLC19A2* gene is highly expressed in the inner ear cells, β-islet cells, and hematopoietic stem cells; therefore, these cells are extremely affected by the lack of THTR-1. Consequently, the typical clinical triad of TRMA manifests: diabetes, megaloblastic anemia, and sensorineural hearing loss. Besides the clinical triad, patients with TRMA have been reported to have additional symptoms, such as visual impairment, congenital heart disease (organic change and/or arrhythmia), short stature, cerebrovascular events, thrombocytopenia, and leukopenia [5].

The β-islet cells in patients with TRMA cannot transport thiamine, and therefore cannot carry out aerobic metabolism effectively. This results in β-cell loss and reduction in insulin secretion, leading to type I diabetes even though the patient does not have β-cell autoimmunity. In some patients, C-peptide levels are maintained for a long time [6]. Thiamine therapy enables most patients to stop insulin administration or reduce the dosage [7]. Nevertheless, some patients may need insulin administration during puberty because of accelerated apoptosis of β-cells, necessitating long-term follow-ups [8]. Development of diabetes into ketoacidosis is rare in patients with TRMA; however, in thiamine shortage, ketoacidosis may also appear before puberty [9].

The activity of transketolase in hematopoietic cells in the bone marrow decreases in patients with TRMA syndrome due to the lack of thiamine. This, in turn, can cause several cellular abnormalities, such as disorders in ribosomal and DNA synthesis, immature nucleus formation, and hypertrophy of the cell. Consequently, blood cell formation decreases, leading to anemia. Additionally, thiamine shortage can also affect the synthesis of ferroprotoporphyrin and result in the formation of ring sideroblasts [10–12]. Hitherto, only one case without anemia has been reported [13]. Hemoglobin levels of most patients with TRMA syndrome can return to normal levels following thiamine treatment.

Patients with TRMA syndrome are usually congenitally deaf or have hearing loss, which gradually worsens. Liberman et al. [14] found in rats that low thiamine diet (2 mg thiamine/kg) could be detrimental to cochlear inner hair cells (IHCs) and cause their atrophy. This observation supports the hypothesis that early intervention with thiamine treatment may antagonize progression of deafness in patients with TRMA syndrome. However, according to Akin et al. [15], such treatment does not suffice to stop the hearing loss. In fact, the majority of the patients with TRMA syndrome still had deafness, even with thiamine therapy. Therefore, it is generally believed that thiamine therapy cannot alleviate deafness. Nevertheless, it is necessary to test whether increased doses of thiamine, or an earlier onset of the treatment can prevent hearing loss altogether or decelerate its progression.

In his 2016 review, Ortigoza-Escobar et al. [5] list several other symptoms observed in addition to the clinical triad, such as visual impairment (retinitis pigmentosa [16], optic atrophy [17], photoreceptor degeneration [18], macular lesions [4] and nystagmus [19]), congenital heart disease (atrial arrhythmia, or atrial septal defect [5] are frequent), short stature [5] [20], cerebrovascular disorders (stroke and epilepsy [4]), thrombocytopenia, and leukopenia. Furthermore, these authors also mention that only one TRMA patient presented 1–2 symptoms in addition to the triad despite he had a homozygous mutation, whereas three patients had all the symptoms mentioned above. Notably, the patient we describe here had all the symptoms mentioned above, even though he had a heterozygous mutation. His clinical presentation was more severe than the previously reported cases, For instance, he had ketoacidosis at the early age of 6 years old, and presented all the known symptoms. Furthermore, he had cerebral infarction, paralysis of his left arm, and ventricular premature beat (VPB) when he was 9 months old, This is the first time such phenotype has been reported.

Currently, 45 different mutations are documented in the *SLC19A2* gene from TRMA patients according to the HGMD database, most of which are from consanguineous backgrounds [5]. Most of these mutations are missense/nonsense mutations, which introduce a premature stop codon, or frameshift mutations. Only a few are missense mutations, which change the amino acid residue, causing abnormalities in the protein structure [4, 12]. The patient presented here was found to have a novel compound heterozygote mutation in the *SLC19A2* gene, comprising a duplication (c.405dupA) on one allele, and a single nucleotide deletion (c.903delG) on the other allele. Alamut functional software predicted that the mutation could lead to premature translational termination, which can cause formation of a truncated protein, and thus, the mutation was classified as a “pathogenic variation.”

Thiamine supplementation is the main treatment of TRMA syndrome. In most reported cases, thiamine therapy alleviated diabetes and anemia [4], except for only one case in which diabetes did not improve [20]. However, Some patients again became diabetic and anemic after stopping thiamine therapy [19, 21]. Lifelong therapeutic use of thiamine at the range of 25–75 mg/day was usually recommended, with earlier start of the therapy being more effective [7]. In all the reported cases, the dosage was increased up to 300 mg/day [5]. In the case presented here, the dose was only 30 mg/day, and the follow-up exams showed that diabetes, anemia, and arrhythmia of the patient were ameliorated.

In summary, here we report a new case of TRMA syndrome in a patient descended from non-consanguineous Chinese parents. A novel compound heterozygote mutation in the *SLC19A2* gene comprising a duplication (c.405dupA) and a nucleotide deletion (c.903delG) was identified. This is the first time a severe phenotype with cerebral infarction and VPB has been reported in patients with TRMA syndrome. The clinical symptoms of TRMA syndrome can manifest anytime between infancy and adolescence. A misdiagnosis during this short time can be detrimental, leading to a significant delay between the onset of the symptoms and an accurate diagnosis. Presentation of the clinical triad, as well as laboratory examination and genetic analysis are instrumental in confirming the diagnosis.

## Data Availability

The data and materials used during the current study are available from the first author on reasonable request.

## Abbreviations

ECG: Electrocardiogram
GA: Glycated albumin
GADA: Glutamic acid decarboxylase antibody
HbA1c: Glycosylated hemoglobin
IAA: Insulin antibody
ICA: Islet cell antibody
IHCs: Inner hair cells
PDHC: Pyruvate dehydrogenase complex
TDP: Diphosphate
THTR: The high affinity thiamine transporter
TK: Transketolase
TRMA: Thiamine-responsive megaloblastic anemia
VPB: Ventricular premature beat
α–KGDH: α-ketoglutarate dehydrogenase
